# Fisetin Inhibits UVA-Induced Expression of MMP-1 and MMP-3 through the NOX/ROS/MAPK Pathway in Human Dermal Fibroblasts and Human Epidermal Keratinocytes

**DOI:** 10.3390/ijms242417358

**Published:** 2023-12-11

**Authors:** Hye-Yeon Jang, Gi-Beum Kim, Jeong-Mi Kim, Sang Yull Kang, Hyun-Jo Youn, Jinny Park, Su Yeon Ro, Eun-Yong Chung, Kwang-Hyun Park, Jong-Suk Kim

**Affiliations:** 1 Department of Biochemistry and Molecular Biology, Institute for Medical Sciences, BK21FOUR 21st Century Medical Science Creative Human Resource Development Center, Jeonbuk National University Medical School, Jeonju 54907, Republic of Korea; janghyeyeon@naver.com (H.-Y.J.); biomedico@naver.com (G.-B.K.); wjdal4219@hanmail.net (J.-M.K.); 2 Infectious Diseases Therapeutic Center, Korea Research Institute of Chemical Technology (KRICT), Daejeon 34114, Republic of Korea; 3 Department of Surgery, Research Institute of Clinical Medicine, Jeonbuk National University Hospital, Biomedical Research Institute, Jeonbuk National University, Jeonju 54907, Republic of Korea; sykang@jbnu.ac.kr (S.Y.K.); yhj0903@jbnu.ac.kr (H.-J.Y.); 4 Department of Medical Oncology and Hematology, Ansan Hospital, Korea University College of Medicine, Ansan 15355, Republic of Korea; jhagnes@hananet.net; 5 Department of Anesthesiology and Pain Medicine, Bucheon St. Mary’s Hospital, College of Medicine, The Catholic University of Korea, Bucheon 14647, Republic of Korea; suynm11@naver.com (S.Y.R.); anes36@catholic.ac.kr (E.-Y.C.); 6 Department of Emergency Medical Rescue, Nambu University, Gwangju 62271, Republic of Korea; 7 BioMedical Science Graduate Program (BMSGP), Department of Emergency Medicine, Chonnam National University, Hwasun 58128, Republic of Korea

**Keywords:** fisetin, ultraviolet A, matrix metalloproteinase, NADPH oxidase, reactive oxygen stress

## Abstract

Fisetin is a flavonoid found in plants and has been reported to be effective in various human diseases. However, the effective mechanisms of ultraviolet-A (UVA)-mediated skin damage are not yet clear. In this study, we investigated the protective mechanisms of fisetin regarding UVA-induced human dermal fibroblasts (HDFs) and human epidermal keratinocytes (HEKs) damages. Fisetin showed a cytoprotective effect against UVA irradiation and suppressed matrix metalloproteinases (MMPs), MMP-1, and MMP-3 expression. In addition, fisetin was rescued, which decreased mRNA levels of pro-inflammatory cytokines, reactive oxygen species production, and the downregulation of MAPK/AP-1 related protein and NADPH oxidase (NOX) mRNA levels. Furthermore, UVA-induced MMP-1 and MMP-3 were effectively inhibited by siRNAs to NOX 1 to 5 in HDFs and HEKs. These results indicate that fisetin suppresses UVA-induced damage through the NOX/ROS/MAPK pathway in HDFs and HEKs.

## 1. Introduction

The human skin is the only organ directly exposed to ultraviolet (UV) light. Skin damage, an effect of UV-induced skin damage, is unavoidable in human life. The adverse effects of sunlight UV on the skin are thus well known [1,2,3,4,5,6]. However, there are no effective protective strategies against UV-induced skin damage.

Skin damage is the effect of chronic exposure to UV rays (photoaging), and this is mostly seen on the skin of farmers and fishermen, as they are frequently exposed to UV rays. Externally, the skin is rough and has face rhytids; thus, characteristic rhombic rhytids can be seen on the nape. The histological changes of photoaging skin include increased thickness of the epidermis and abnormal hyperactivity of melanocytes [4]. Elastic fibers, which are the main components of the dermis, proliferate, ideally with the expansion of dermal capillaries [7]. The wavelength range that causes photoaging is not known, but it is known that both ultraviolet-A (UVA) and ultraviolet-B (UVB) rays, which penetrate deep into the dermis, are involved [6].

Ultraviolet ray-induced skin damage is a very complex process and is associated with a variety of factors, such as cytokines and reactive oxygen species (ROS) [5,8,9]. In addition, UVB exposure directly affects DNA damage [6]. Repetitive exposure to UVB increases intracellular ROS, leading to oxidative DNA damage and activation of inflammation and extracellular matrix (ECM)-remodeling proteins, including matrix metalloproteinases (MMPs) [5,6,10]. Particularly, the UVB-induced cellular damage mechanism in epidermal keratinocytes explains IL-1b secretion through cross-talk between Ca^2+^ and reactive oxygen species, providing insight toward potential targets against inflammatory responses via CD38 signaling [11], while investigations of protective mechanisms against UVA-induced damage have not been reported in human dermal fibroblasts (HDF) and human epidermal keratinocytes (HEK), parallelly.

Therefore, several materials that inhibit photoaging have been studied, mainly focusing on the role of UVB protection and antioxidants [10,12]. Among living beings in nature, plants cannot avoid sunlight because they conduct photosynthesis; thus, they are always exposed to UVB. The ability of plants to resist the damage caused by UVB radiation can be attributed to flavonoids, which are universally present in leaves [13]. Moreover, flavonoids have attracted attention as anti-photoaging agents because they act as excellent scavengers against various oxidizing species [12,13].

Fisetin is a dietary flavonoid and a natural polyphenolic compound found in many vegetables and fruits, such as strawberries, apples, and grapes. It was discovered by the Austrian chemist Josef Herzig in 1891 and has been extensively studied in many fields. The antioxidant and antitumor effects of fisetin are well known [14,15,16].

A previous study showed that fisetin inhibits the invasion of breast cancer cells by controlling MMPs [17]. More recently, fisetin has also been reported to be important for maintaining skin health. Chiang et al. reported that fisetin inhibits the phosphorylation of UVB-induced mitogen-activated protein kinases (MAPKs) and increases the activity of MMPs in skin fibroblasts [18,19]. However, the mechanism of UV-induced skin damage is not yet fully understood.

This study aimed to investigate the role of fisetin in the regulation of UV-induced MMP-1/3 expression in human skin fibroblasts (HDFs) and human epidermal keratinocytes (HEKs).

## 2. Results

### 2.1. The Cytoprotective Effect of Fisetin and Inhibitory Effect of Fisetin on UVA-Induced MMP-1 and MMP-3 Expression

**Figure 1:**
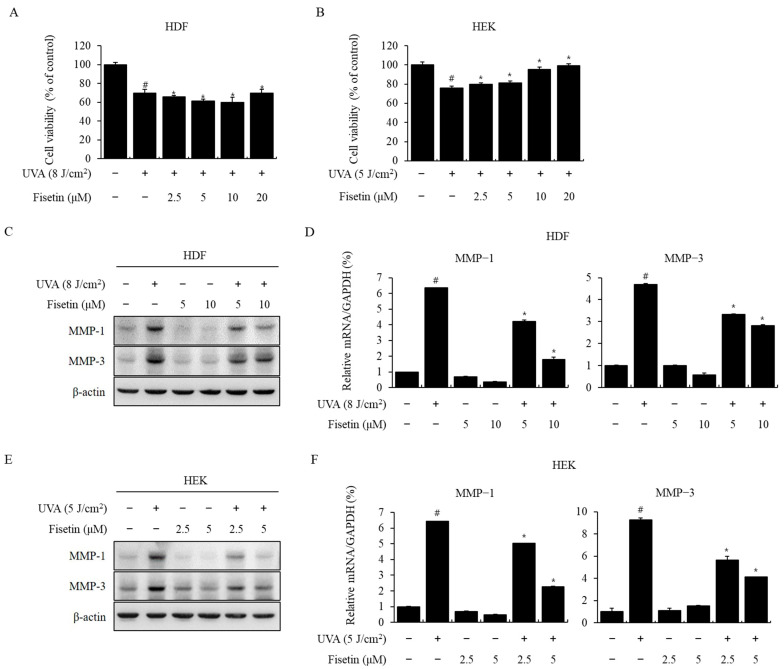
Effects of fisetin on HDF and HEK viability. Cells were treated with fisetin at various concentrations, and cytotoxicity was evaluated after 24 h. (**A**,**B**) After pretreatment with fisetin, cells were irradiated with UVA and incubated for 24 h. Cell viability was analyzed using the MTT assay. Fisetin inhibited UVA-induced MMP-1 and MMP-3 expression in HDFs and HEKs. HDFs and HEKs were treated with fisetin, irradiated with UVA, and then incubated for 24 h. (**C**,**E**) MMP-1 and MMP-3 protein expression was analyzed using Western blotting; β-actin was used for loading control. (**D**,**F**) MMP-1 and MMP-3 mRNA levels were analyzed using real-time PCR; GAPDH was used for internal control. The data are expressed as the means ± SE (n = 3). # *p* < 0.01 vs. untreated control, * *p* < 0.01 vs. UVA.

The cytotoxicity of fisetin on HDFs and HEKs was assessed using the MTT assay. HDFs and HEKs were treated with fisetin at 2.5, 5, 10, and 20 μM for 24 h. Both cells treated with fisetin showed no cytotoxicity at concentrations less than 10 μM. The viability of HDFs and HEKs decreased similarly to 75% when irradiated with UVA. Pretreatment with fisetin in HDFs had no significant dose-dependent recovery effect in this assay model (Figure 1A), but treatment with 5, 10, and 20 μM fisetin increased the survival rate to 82%, 95%, and 99% (Figure 1B), with no significant morphological changes (Supplementary Figure S1). These results indicate that fisetin has a cytoprotective effect on cell viability that is decreased by UVA irradiation.

To investigate the effect of fisetin on UVA-induced expression of MMP-1 and MMP-3, HDFs and HEKs were irradiated with UVA and treated with fisetin at each concentration for 24 h. After HDFs and HEKs were treated with UVA, the expression of MMP-1 and MMP-3 (protein and mRNA) was measured using Western blotting and RT-qPCR. UVA irradiation induced MMP-1 and MMP-3 expression in a dose-dependent manner, which was significantly reduced by fisetin (Figure 1C–F). These results showed that fisetin effectively inhibited UVA-induced expression of MMP-1 and MMP-3 in both cell lines.

### 2.2. Fisetin Inhibited UVA-Induced Inflammatory Cytokine Expression and ROS

**Figure 2:**
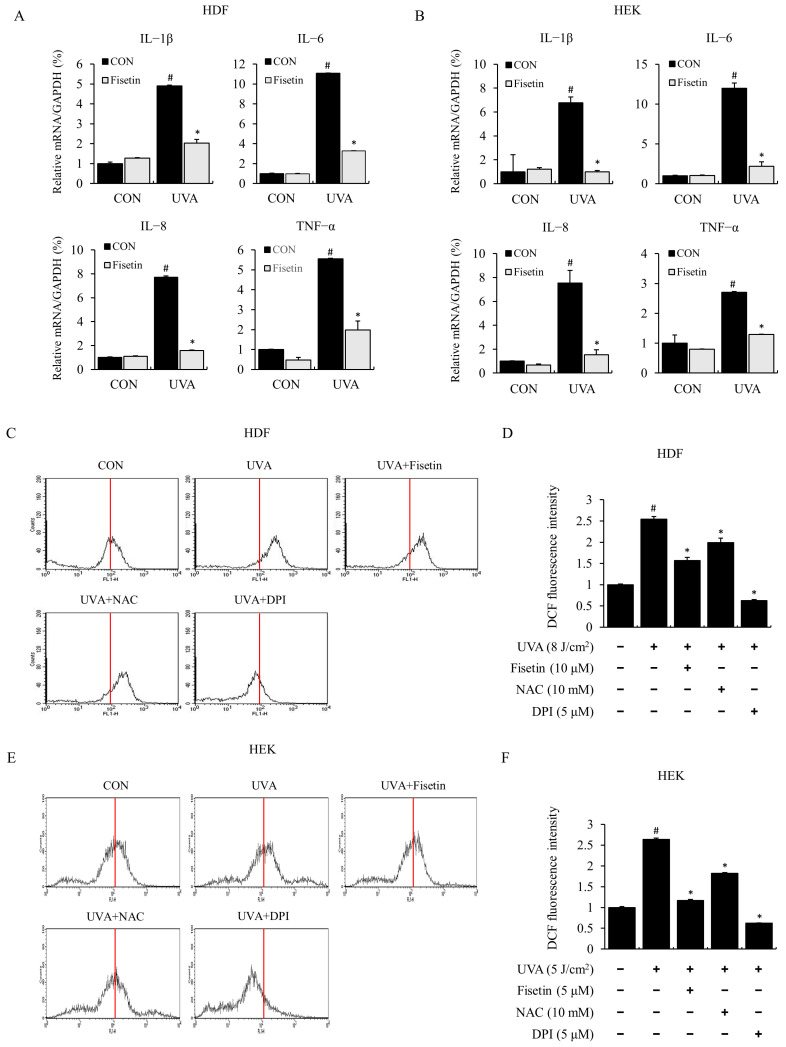
Fisetin inhibited the expression of pro-inflammatory cytokines in UVA-irradiated HDFs and HEKs. (**A**) HDFs were treated with fisetin (10 μM) and exposed to UVA (8 J/cm^2^). (**B**) HEKs were treated with fisetin (5 μM) and exposed to UVA (5 J/cm^2^). After 4 h, pro-inflammatory cytokine (IL-1β, IL-6, IL-8, and TNF-α) mRNA levels were analyzed using real-time PCR. GAPDH was used as an internal control. (**C**–**F**) Inhibitory effects of fisetin on UVA-induced reactive oxygen species (ROS) production in HDFs (**C**,**D**) and HEKs (**E**,**F**). HDFs and HEKs were treated with fisetin, irradiated with UVA, and then incubated for 1 h. Cells were then washed with PBS and incubated with DCF-DA for 30 min at room temperature. Intracellular ROS production was measured using DCF-DA fluorescence using a FACS flow cytometer. The data are expressed as the means ± SE (n = 3). # *p* < 0.01 vs. untreated control, * *p* < 0.01 vs. UVA.

Long-term exposure of human skin to ultraviolet radiation (UVR) in sunlight results in skin damage, such as sunburn. UV rays are known to induce skin inflammatory responses in the skin by inducing the synthesis and release of pro-inflammatory cytokines such as tumor necrosis factor-alpha (TNF-α) and interleukin 6 (IL-6) [9]. In this study, we measured UVA-induced inflammatory cytokines and mRNA expressions using real-time PCR in HDFs and HEKs. The expression of inflammatory cytokines, such as IL-1β, IL-6, IL-8, and TNF-α, was increased by UVA irradiation and markedly reduced by fisetin pretreatment (Figure 2A,B). These results show that fisetin exhibits anti-inflammatory effects by inhibiting the production of UVA-induced pro-inflammatory cytokines.

To investigate the effect of fisetin on UVA-induced ROS production, ROS production was measured using the DCF-DA fluorescence method. When HDFs and HEKs were exposed to UVA, ROS production was increased compared to that in the control, and ROS production was inhibited by fisetin. In this study, we demonstrated that ROS production was decreased by the ROS inhibitor NAC and the NOX inhibitor DPI as positive controls (Figure 2C–F). Similarly, regulatory effects of fisetin, NAC, and DPI on UVA-induced ROS production in intracellular regions were observed through fluorescence microscopic imaging (Supplement Figure S2). These results indicated that fisetin significantly blocked UVA-induced ROS production in HDFs and HEKs, indicating that NOX was involved in UVA-induced ROS production.

### 2.3. Fisetin Inhibited UVA-Induced MAPK/AP-1 Activation

**Figure 3:**
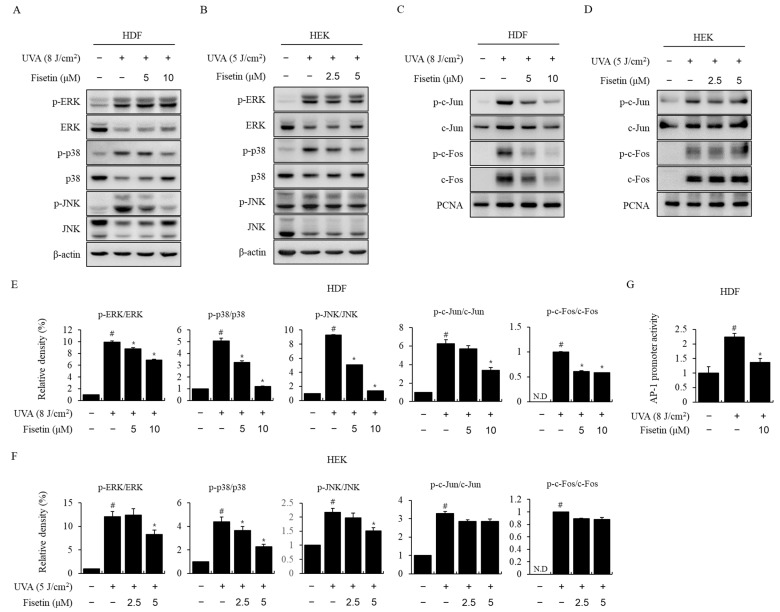
Fisetin suppressed UVA-induced MAPK activation in HDFs and HEKs. (**A**,**B**) Fisetin inhibits UVA-induced AP-1 activation in HDFs. (**C**,**D**) HDFs and HEKs were treated with fisetin and exposed to UVA. After 30 min, total ERK, p38, JNK and phospho-ERK, phospho-p38, and phospho-JNK levels were analyzed using Western blotting. β-actin was used for loading control. After 2 h, nuclear extracts were prepared. Western blotting was performed to determine the nuclear levels of the AP-1 (p-c-Jun, p-c-Fos) subunits. PCNA was used as an internal control for the nuclear fraction. (**E**,**F**) To assess phosphorylation levels, the density of each band was quantified using ImageJ software (ver. 1.52 for MS Windows), and the relative density ratio of each protein was calculated accordingly. To assess phosphorylation levels, the density of each band was quantified using the ImageJ software (ver. 1.52 for MS Windows), and the relative density ratio of each protein was calculated accordingly. (**G**) HDFs were treated with fisetin and irradiated with UVA, and the promoter activity of AP-1 was measured using the dual-luciferase reporter assay. The data are expressed as the means ± SE (n = 3). # *p* < 0.01 vs. untreated control, * *p* < 0.01 vs. UVA. ND: not detected.

To identify the signaling pathway that suppresses MMP-1 and MMP-3 expression because of fisetin, the effect of fisetin on UVA-induced MAPK activation was investigated using Western blotting. In HDFs and HEKs, UVA induced ERK, P38, and JNK phosphorylation, whereas fisetin treatment inhibited P38 and JNK phosphorylation in a concentration-dependent manner (Figure 3A,B). The effect of fisetin on AP-1 activation was investigated using a Western blotting and luciferase assay. In HDFs, fisetin reduced the UVA-induced nuclear migration of p-c-Jun and p-c-Fos (Figure 3C). However, fisetin had no significant effect on AP-1 activation in HEKs (Figure 3D). Statistically, pretreatment of fisetin significantly modulated the UVA-induced cellular signaling pathway in HDF (Figure 3E) and HEK (Figure 3F) in a dose-dependent manner. Moreover, luciferase analysis showed that fisetin also suppressed the UVA-induced increase in AP-1 promoter activity in HDF (Figure 3G). These results indicated that fisetin suppressed UVA-induced MMP-1 and MMP-3 expression through the regulation of the MAPK/AP-1 pathway.

### 2.4. Effects of Fisetin on NOX Signaling Pathway and MMP-1, 3 Expression

**Figure 4:**
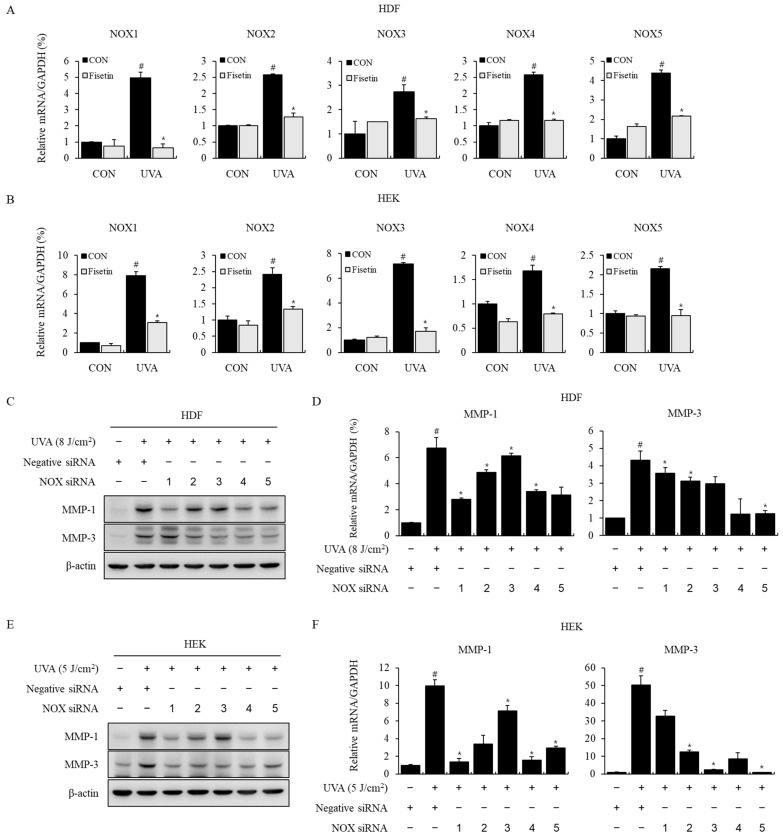
Fisetin inhibited the expression of NOX in UVA-irradiated HDFs and HEKs. (**A**) HDFs were treated with fisetin (10 μM) and exposed to UVA (8 J/cm^2^). (**B**) HEKs were treated with fisetin (5 μM) and exposed to UVA (5 J/cm^2^). NOX1–NOX5 mRNA levels were analyzed using real-time PCR. GAPDH was used as an internal control. The data are expressed as the means ± SE (n = 3). # *p* < 0.01 vs. untreated control, * *p* < 0.01 vs. UVA. Regulation of MMP-1 and MMP-3 expression by NOX. NOX1–NOX5 were knocked down in HDFs and HEKs using NOX siRNA. Then, the cells were irradiated with UVA and incubated for 24 h. (**C**–**F**) * *p* < 0.01 vs. negative siRNA UVA-treated group. The data are expressed as the means ± SE (n = 3).

We investigated whether fisetin is involved in NOX signaling by establishing that UVA-induced increased intracellular ROS production was suppressed when treated with a NOX inhibitor, DPI. When HDFs and HEKs were exposed to UVA, the increase in mRNA expression of NOX1–NOX5 was determined using real-time PCR, and the responses were suppressed using fisetin (Figure 4A,B).

To determine whether NOX is involved in UVA-induced MMP-1 and MMP-3, NOX siRNA was used to knock down NOX1–NOX5 expression and was measured using Western blotting and real-time PCR. In HDFs, the UVA-induced protein expression of MMP-1 was inhibited when NOX1, NOX4, and NOX5 were knocked down, and protein expression of MMP-3 was significantly inhibited when NOX3, NOX4, and NOX5 were knocked down. MMP-1 mRNA levels were also decreased when NOX1, NOX4, and NOX5 were knocked down, and MMP-3 mRNA levels were significantly suppressed when NOX3, NOX4, and NOX5 were knocked down (Figure 4C,D). In HEKs, UVA-induced MMP-1 protein expression was suppressed when NOX1, NOX2, NOX4, and NOX5 were knocked down, and MMP-3 protein expression was decreased by NOX1–NOX5 siRNA. MMP-1 and MMP-3 mRNA levels were also significantly decreased by all NOX1–NOX5 siRNAs (Figure 4E,F).

## 3. Discussion

ROS, a by-product of normal cellular oxidation, accumulate with cellular degeneration and play an important role in skin damage [8,20]. Aging is thus associated with a conscious increase in oxidative stress, indicating that ROS may play an important role in stimulating degeneration. Indeed, the reduction in ROS production via dietary strategies can extend the lifespan in a variety of animal models. Recent studies have shown that dietary phytochemicals can extend the lifespan [21]. Antioxidants have been used to protect the body from UV-containing chemical compounds. Fisetin, a natural flavonoid, has been shown to inhibit inflammation and cell damage induced by UVB irradiation in various tissues, leading to cataracts and skin cancer; it was also shown to reduce UVB damage to keratinocytes, fibroblasts, and inflammatory cells [19,22,23,24], but research on UVA is still limited. Therefore, we investigated the effect of fisetin on UVA-induced skin damage. The results of this study demonstrate that fisetin has an inhibitory effect on UVA-induced MMP activity and oxidative stress in HDFs and HEKs and has a strong anti-inflammatory effect and a strong effect on NOX inhibition. These findings indicate a relationship between NOX and MMP in fibroblasts and keratinocytes, suggesting an important role for NOX in skin damage. Here, we demonstrated that fisetin could be developed as a potential drug for the prevention of skin damage.

It is well known that low concentrations of flavonoids protect cells, whereas high concentrations cause DNA damage and cell death [25]. We determined that low concentrations of fisetin did not cause cytotoxicity in HDFs and HEKs and that fisetin had a cytoprotective effect against UVA-induced damage in HEKs (Figure 1 and Supplementary Figure S1). Prolonged exposure to UVA is considered to be a major cause of skin damage due to its ability to penetrate the dermal layer, where it binds to the skin collagen [20]. We confirmed that UVA irradiation induced the expression of MMP-1 and MMP-3, which play an important role in ECM homeostasis in HDFs and HEKs, and MMP-1 and MMP-3 expression in fisetin-treated cells was reduced (Figure 1).

It has been reported that ROS are generated in response to UV irradiation and are regulated by various pathways, such as MAPK, to activate transcription factors such as AP-1 and are involved in the expression of pro-inflammatory cytokines and MMPs [8,9,10,26,27]. Various cytokines are known to be involved in the immunological regulation of the skin, including skin inflammation [28,29]. In this study, UVA irradiation in HDFs and HEKs increased the mRNA expression of pro-inflammation-related genes, namely, IL-1β, IL-6, IL-8, and TNF-α, expression of which was significantly decreased in fisetin-treated cells (Figure 2). In addition, fisetin inhibited the UVA-induced phosphorylation of MAPK proteins (ERK, JNK, and p38) and decreased the translocation of AP-1 transcription factors p-c-Jun and p-c-Fos to the nucleus (Figure 3). AP-1 promoter activity was also suppressed in HDFs (Figure 3G). Although superoxide products, including H_2_O_2_, are produced in chemical- or physical energy-induced oxidative stress-related experiments in vivo and in vitro, we used relative changes with instrumental values instead of calculated concentration. So, critical concentrations of ROS and/or H_2_O_2_ are necessary for further UVA-induced damage-related studies.

In this study, UVA irradiation markedly and consistently altered MAPK and AP-1 pathway component molecules. We confirmed and assured the statistics analysis that fisetin is effective in rescuing damage signaling. As per the reviewer’s comments, we described this in the Discussion section. We showed that UVA irradiation increased ROS production in HDFs and HEKs and that fisetin pretreatment inhibited ROS production increased by UVA. Similarly, the ROS inhibitor (NAC) and the NOX inhibitor (DPI) also abrogated ROS production (Figure 2 and Supplementary Figure S2). Interestingly, ROS production was increased more by UV in cells pretreated with fisetin than in cells treated with NAC. These results suggest that fisetin regulates the expression of pro-inflammatory genes via ROS and MAPK pathways in HDFs and HEKs.

NOX is closely related to ROS generation [30,31,32,33], and many studies related to UV and NOX have been reported [34,35,36]. However, studies showing a direct association between NOX and MMP have not yet been reported. In this study, we investigated the relationship between fisetin and NOX by demonstrating that fisetin showed an excellent inhibitory effect, like DPI, a NOX inhibitor, on the increase in ROS production induced by UVA irradiation. When HDFs and HEKs were irradiated with UVA, NOX1–NOX5 mRNA levels were increased, and all NOX1–NOX5 mRNA levels were suppressed by fisetin (Figure 4). To demonstrate the role of NOX in UVA-induced MMP expression, NOX was knocked down using NOX siRNA. When NOX was knocked down using NOX1–NOX5 siRNA, the mRNA levels and protein expression of MMP-1 and MMP-3 increased by UVA irradiation were decreased by NOX siRNA (Figure 4). These results emphasize the important role of NOX in UVA-induced MMP-1 and -3 expression in HDFs and HEKs, and fisetin can be used as a strong inhibitor of the NOX pathway as it has a specific inhibitory effect on UVA-induced NOX. Further studies are needed to elucidate the mechanism by which fisetin inhibits NOX.

The general public assumes dermal health maintenance is an important factor in personal satisfaction. People spend efforts on dermal care techniques, including single/combined materials, to make their dermal condition look healthier. Topical and/or oral materials are approved in the care of ultraviolet-induced skin malfunctions as drugs and non-drugs, while anti-inflammatory drugs and their ointments are used for the treatment of several skin damages. However, all these treatments have their own limitations, and almost every user shows various needs for safe, natural-product-based materials. The protective mechanism of fisetin in UVA-induced damage on epidermal regions has been unclear in the past, but we newly proved here that it could be through its antioxidant properties with the regulation of MMPs expressions and pro-inflammatory cytokine production via the NOX/ROS/MAPK pathway. Furthermore, a comparative analysis of fisetin with UVA-induced skin damage with non-UVA-induced skin damage is required for further studies.

In addition, fisetin is reported to bind to GSK-3β in melanoma cells and zebrafish and induce melanin production, which protects cells from ultraviolet rays in the current molecular docking study [37]. However, docking studies on UV protection of fisetin in human dermal fibroblasts (HDF) and human epidermal keratinocytes (HEK) have not been reported. Therefore, it is necessary to conduct a docking study of fisetin on the target protein and conduct additional research on the interaction.

**Figure 5:**
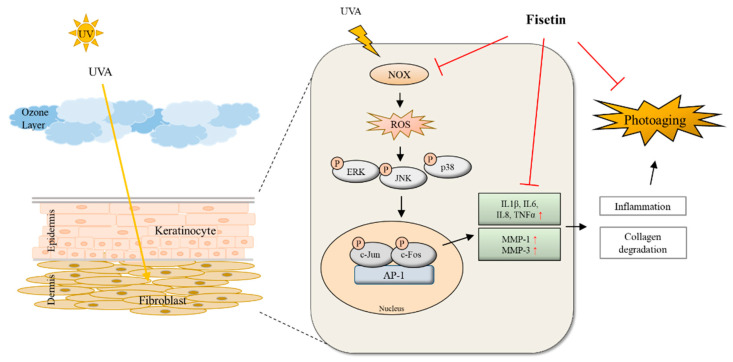
Schematic diagram of protective mechanisms of the fisetin on UVA-induced HDF and HEK cell damages via MMP-1 and MMP-3 expression through the NOX/ROS/MAPK pathway.

In conclusion, we demonstrated that fisetin, a natural flavonoid, suppressed MMP-1 and MMP-3 expression through the NOX–ROS pathway in UVA-irradiated HDFs and HEKs and inhibited pro-inflammatory gene expression, leading to anti-inflammatory effects. Moreover, the phosphorylation of MAPKs and AP-1 proteins ERK, P38, and JNK was decreased in a dose-dependent manner, and the translocation of p-c-Jun and p-c-Fos to the nucleus was inhibited (Figure 5). The results of this study suggest that fisetin could be developed as a potential drug for the prevention of skin damage, and NOX regulation by fisetin could be an important indicator of MMP activity.

## 4. Materials and Methods

### 4.1. Materials

Fisetin was obtained from Sigma-Aldrich (St. Louis, MO, USA). Phosphate-buffered saline (PBS), fetal bovine serum (FBS), Medium 154, and human keratinocyte growth supplement were obtained from Gibco-BRL (Gaithersburg, MD, USA). Antibodies against MMP-1, MMP-3, p-c-Jun (Abcam, Cambridge, UK), PCNA, horseradish peroxidase (Santa Cruz Biotechnology, Dallas, TX, USA), p-ERK, ERK, p-P38, P38, p-JNK, JNK, c-Jun, p-c-Fos, c-Fos (Cell Signaling Technology, Beverly, MA, USA), and β-actin (Sigma-Aldrich, St. Louis, MO, USA) were used for Western blotting.

### 4.2. Cell Culture

Human dermal fibroblasts (HDFs) were purchased from GIBCO (Invitrogen, Carlsbad, CA, USA) and grown in Hepes-bicarbonate-buffered Dulbecco’s modified Eagle’s medium supplemented with 10% FBS and 1% antibiotics at 37 °C in a 5% CO_2_ incubator. HEK was purchased from Thermo Fisher Scientific (Waltham, MA, USA) and grown in Medium 154 supplemented with 10% FBS, 1% antibiotics, and 1% HEK growth supplement at 37 °C in a 5% CO_2_ incubator.

### 4.3. UV Irradiation

Cells were rinsed twice with PBS before UVA irradiation and irradiated with UV CL-508M UV cross-linker using a UVA T-8L lamp (Vilber Lourmat, Paris, France). Immediately after irradiation (for 27 min for 5 J/cm^2^ and 40 min for 8 J/cm^2^) (λmax, 365 nm, no detectable emissions below 320 nm), a fresh medium was added to the cells, and the response was measured after incubation for each experimental condition. Control cells received the same medium change but were not exposed to UVA radiation.

### 4.4. Cell Viability Assay

Cell viability was performed as described in Panjapa [38] using a 3-[4,5-dimethylthiazol-2-yl]-2,5-diphenyltetrazolium bromide MTT assay (Sigma-Aldrich, St. Louis, MO, USA). Cells were seeded at a density of 6 × 10^3^ cells in 96-well plates and incubated at 37 °C for 24 h. When cells reached 70–80% confluence, they were pretreated with fisetin (2.5, 5, 10, or 20 μM) for 4 h, irradiated with UVA (5 J/cm^2^ or 8 J/cm^2^), and incubated for 24 h. The cells were then washed with PBS and incubated with the MTT solution (0.5 mg/mL) for 4 h at 37 °C. Following incubation, formazan crystals were dissolved in DMSO (100 μL/well), and the optical density was measured using a microplate reader (Bio-Rad, Hercules, CA, USA) at 570 nm.

### 4.5. Preparation of Nuclear Extract and Western Blotting Analysis

Cytoplasmic and nuclear extracts were prepared using NE-PER^®^ Nuclear and Cytoplasmic Extraction Reagents (Pierce Biotechnology, Rockford, IL, USA). Cells were washed with cold PBS and lysed using M-PER Mammalian Protein Extraction Reagent (Thermo Fisher Scientific, Waltham, MA, USA). Protein samples (10 μg) were separated using SDS-PAGE and transferred to Hybond-polyvinylidene fluoride membranes (GE Healthcare Life Sciences, Pittsburgh, PA, USA) for 1 h. The membranes were blocked for 2 h with 5% skim milk or 5% bovine serum albumin and then incubated with a primary antibody overnight at 4 °C. Then, horseradish peroxidase-conjugated IgG, as a secondary antibody, was added, followed by incubation for 1 h at room temperature. Protein levels were detected using the LAS-4000 image analyzer (FujiFilm, Tokyo, Japan), and their concentrations were determined using the ImageJ software (ver. 1.52 for MS Windows, NIH, Bethesda, MD, USA).

### 4.6. Luciferase Assay

Luciferase assay was performed as described in a previous study [39]. Cells were seeded into 24-well plates and then transfected with an AP-1 reporter plasmid (provided by Professor Chul-Ho Kim, SungKyunKwan University, Suwon, Republic of Korea) using the Lipofectamine 2000 reagent (Invitrogen, Carlsbad, WA, USA). The transfected cells were pretreated with fisetin for 1 h, irradiated with UVA, and then incubated. The luciferase assay was performed using the Dual-Glo^®^ Luciferase Assay System (Promega, Fitchburg, WI, USA) according to the manufacturer’s instructions, and luciferase activity was detected using a luminometer (Lumat LB 9507, EG & G Berthold, Gaithersburg, MD, USA).

### 4.7. Quantification of Intracellular ROS

The intracellular ROS production in HDFs and HEKs was measured using an oxidation-sensitive fluorescent probe dye, DCF-DA. To measure intracellular ROS, the cells were incubated for 30 min at room temperature with 0.5% BSA containing 20 μM DCF-DA (Sigma-Aldrich, St. Louis, MO, USA). DCF fluorescence was detected using a FACS flow cytometer (BD Biosciences, San Jose, CA, USA).

### 4.8. RNA Isolation and Quantitative Reverse Transcription PCR (RT-qPCR)

Total RNA was isolated from cells using the TRIzol reagent (Invitrogen), and cDNA was synthesized from 1 μg of total RNA using the PrimeScript™ RT reagent kit (TaKaRa Bio, Shiga, Japan). RNA concentration and purity were determined by measuring the absorbance at 260/280 nm. Real-time PCR was performed using the SYBR Green and ABI PRISM 7900 sequence detection system (Applied Biosystems, Foster City, CA, USA). Specific primers for each gene are listed in Table 1. Human NADPH oxidase (NOX) primers were purchased from QIAGEN (Table 2). To control for variations in mRNA levels, mRNA concentrations were normalized to the GAPDH housekeeping gene.

### 4.9. siRNA Transfection

Cells were transfected with NOX1–NOX5 siRNA (Bioneer, Daejeon, Korea) using Opti-MEM (Gibco, Billings, MT, USA) and Lipofectamine RNAiMAX (Invitrogen Gaithersburg, MD, USA). Negative control siRNA was purchased from Genotech (Daejeon, Republic of Korea). Experiments were performed according to the Lipofectamine RNAiMAX transfection protocol at a siRNA concentration of 10 nM. The Lipofectamine RNAiMAX-siRNA complex was added to the cells, and after 6 h of transfection, the growth medium was replaced. Cells were irradiated with UVA 24 h after transfection and then used in the experiments. Knockdown of NOX was verified by using RT-qPCR.

### 4.10. Statistical Analyses

Statistical analyses were performed using ANOVA and Duncan’s test. Differences were considered statistically significant at *p* < 0.05.

## Figures and Tables

**Table 1:** Primer sequences used for RT-qPCR analysis of the specified genes (For: forward, Rev: Reverse).

Gene	Primer Sequences	Accession No.
MMP-1	For: AGTGACTGGGAAACCGATGCTGA Rev: GCTCTTGGCAAATCTGGCCTGTAA	NM_001145938
MMP-3	For: ATTCCATGGAGCCAGGCTTTC Rev: CATTTGGGTCAAACTCCAACTGTG	NM_002422
IL-1β	For: TCCTGCGTGTTGAAAGATGATAA Rev: CAAATCGCTTTTCCATCTTCTTC	NM_000576
IL-6	For: TACCCCCAGGAGAAGATTCC Rev: GCCATCTTTGGAAGGTTCAG	NM_000600
IL-8	For: AGACAGCAGAGCACACAAGC Rev: ATGGTTCCTTCCGGTGGT	NM_000584
TNF-α	For: CTGCTGCACTTTGGAGTGAT Rev: AGATGATCTGACTGCCTGGG	NM_000594
GAPDH	For: ATGGAAATCCC ATCACCATCTT Rev: CGCCCCACTTGA TTTTGG	NM_002046

**Table 2:** Primer catalog number used for RT-qPCR analysis of the specified genes.

**Gene**	**Company**	**Catalog No.**	**Accession No.**
NOX1	QIAGEN	PPH06068A	NM_007052
NOX2	QIAGEN	PPH10407A	NM_000397
NOX3	QIAGEN	PPH06074A	NM_015718
NOX4	QIAGEN	PPH06078A	NM_016931
NOX5	QIAGEN	PPH17569A	NM_024505

## Data Availability

Data is contained within the article and Supplementary Materials.

## Supplementary Materials

The supporting information can be downloaded at: https://www.mdpi.com/article/10.3390/ijms242417358/s1.
