# Supplementary material for: Fisetin Inhibits UVA-Induced Expression of MMP-1 and MMP-3 through the NOX/ROS/MAPK Pathway in Human Dermal Fibroblasts and Human Epidermal Keratinocytes
Source: Int J Mol Sci. 2023 Dec 11;24(24):17358. doi: 10.3390/ijms242417358 (PMC10743569; doi:10.3390/ijms242417358)
Supplement: Supplementary file 1 [file ijms-24-17358-s001.zip › ijms-2691853-supplementary.pdf]

Supplementary Data

Article

Fisetin inhibits UVA-induced expression of MMP-1 and MMP-3 through the NOX/ROS/MAPK pathway in human dermal fibroblasts and human epidermal keratinocytes

Hye-Yeon Jang[1,2,†], Gi-Beum Kim[1,†], Jeong-Mi Kim[1], Sang Yull Kang[3], Hyun-Jo Youn[3], Jinny Park[4], Su Yeon Ro[5], Eun-Yong Chung[5], Kwang-Hyun Park[6,7,*] and Jong-Suk Kim[1,*]

[1]Department of Biochemistry and Molecular Biology, Institute for Medical Sciences, BK21FOUR 21st Century Medical Science Creative Human Resource Development Center, Jeonbuk National University Medical School, Jeonju 54907, Korea; janghyeyeon@naver.com (H.-Y.J), biomedico@naver.com (G.-B.K), wjdal4219@hanmail.net (J.-M.K), jsukim@jbnu.ac.kr (J.-S.K).
[2]Infectious Diseases Therapeutic Center, Korea Research Institute of Chemical Technology (KRICT), Daejeon 34114, Korea; janghyeyeon@naver.com (H.-Y.J).
[3]Department of Surgery, Research Institute of Clinical Medicine, Jeonbuk National University Hospital, Jeonbuk National University and Biomedical Research Institute, Jeonju 54907, Korea; sykang@jbnu.ac.kr (S.Y.K), yhj0903@jbnu.ac.kr (H.-J.Y).
[4]Department of Medical Oncology and Hematology, Ansan Hospital, Korea University College of Medicine, Ansan, 15355, Korea; jhagnes@hananet.net (J.P)
[5]Department of Anesthesiology and Pain Medicine, Bucheon St. Mary's Hospital, College of Medicine, The Catholic University of Korea, Bucheon 14647, Korea; suynm11@naver.com (S.Y.R), anes36@catholic.ac.kr (E.-Y.C).
[6]Department of Emergency Medical Rescue, Nambu University, Gwangju 62271, Korea; pkh15129@gmail.com (K.-H.P.).
[7]BioMedical Science Graduate Program (BMSGP), Department of Emergency Medicine, Chonnam National University, Hwasun 58128, Korea.

*Correspondence: khpark@nambu.ac.kr; Tel.: +82-62-970-0220 (K.-H.P.), jsukim@jbnu.ac.kr; Tel.: +82-63-270-3085 (J.-S.K)

†These authors were contributed equally.

Supplementary Figure 1

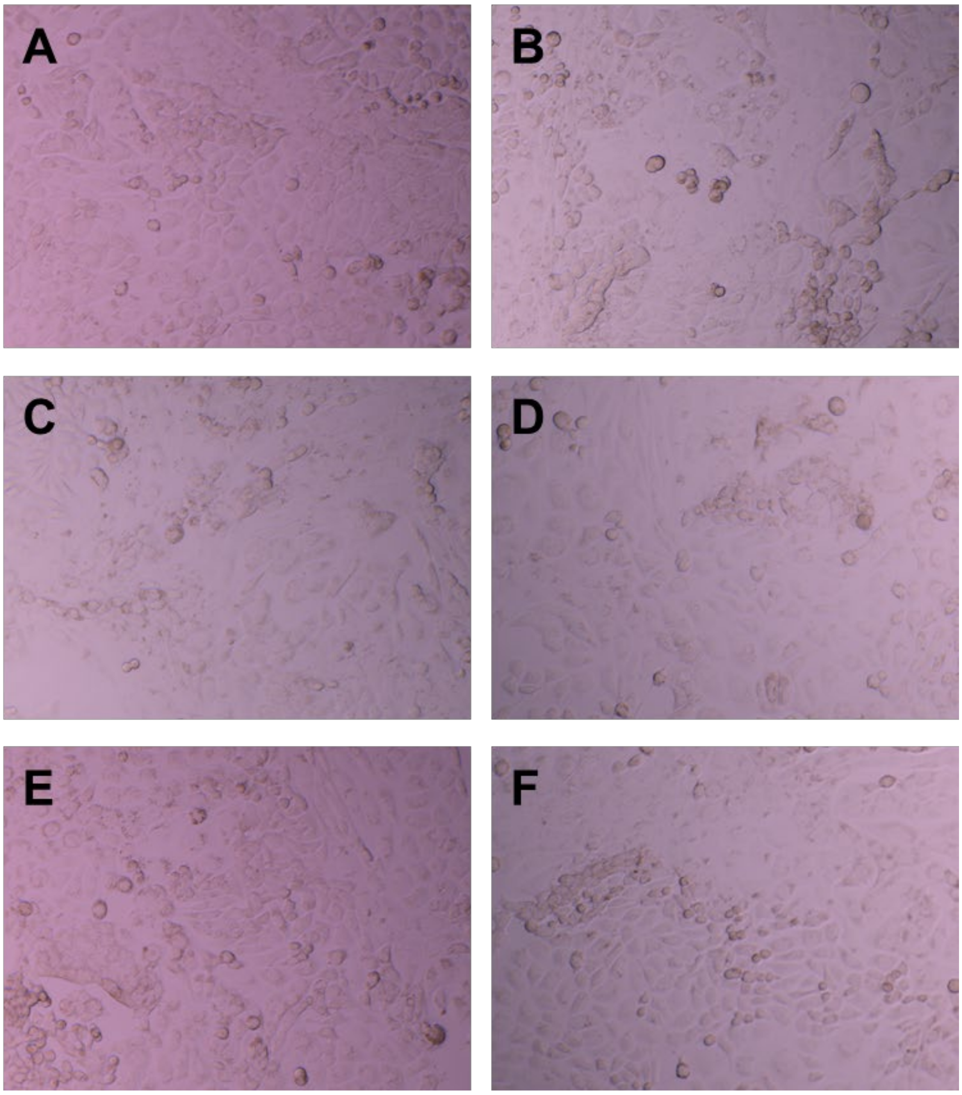

Supplementary Figure S1. Effects of fisetin on HEK viability. Cells were treated with fisetin at various concentrations and cytotoxicity was evaluated after 24 h. After pretreatment with fisetin, cells were irradiated with UVA and incubated for 24 h. Morphological changes of HEK under repetitive doses of UVA. HEK were cultured in a 96-well plate until 80% confluent, and then irradiated with repetitive doses of UVA present or absent fisetin. Following UVA irradiation, HEK and fisetin dose-dependent morphologic changes were observed under a EVOS M5000 Imaging System (Invitrogen, WA, USA). (A) Normal, (B) UVA, (C) UVA + 2.5 μM fisetin, (D) UVA + 5 μM fisetin, (E) UVA + 10 μM fisetin and (F) UVA + 20 μM fisetin. HEK : human epithelial keratinocytes; UVA : ultraviolet A.

Supplementary Figure 2

A HDF

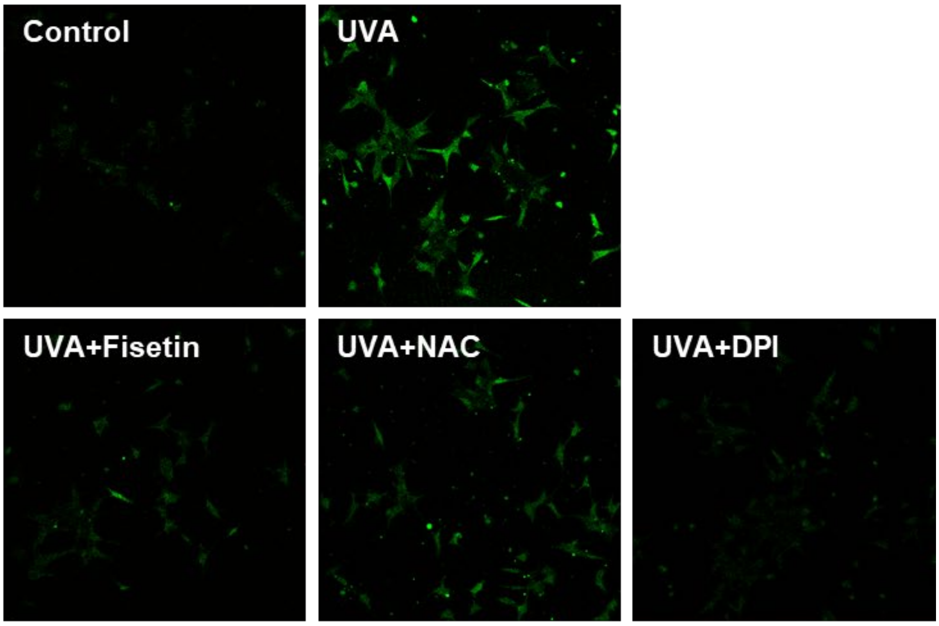

B HEK

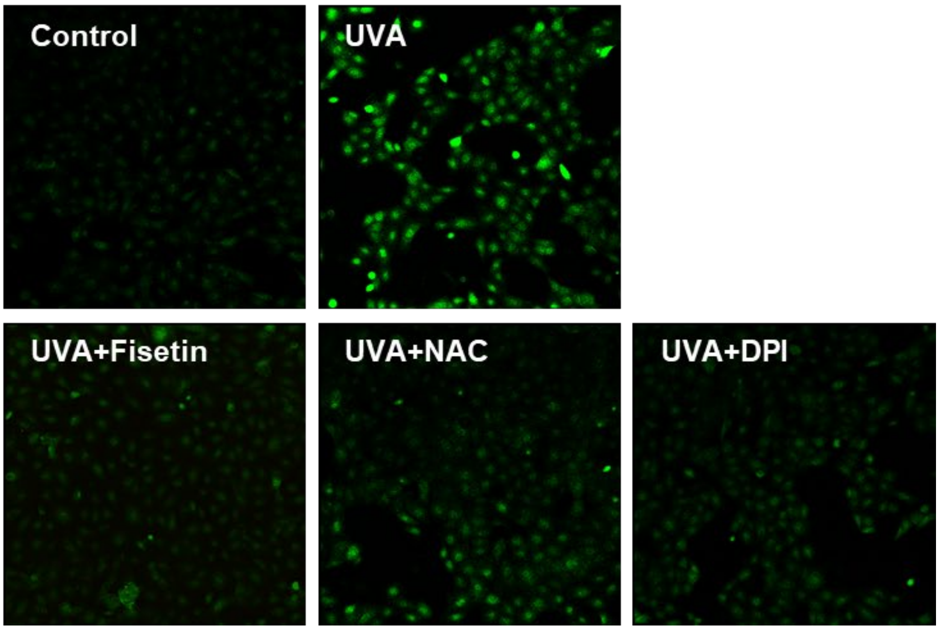

Supplementary Figure S2. Fisetin decreases UVA-induced ROS production in (A) HDF and (B) HEK. Six groups of HDF and HEK were incubated in individual 35 mm dish with conditioned medium, 10 μM fisetin, 10 mM NAC, 5 μM DPI, respectively. These groups were irradiated with 8 J/cm^2 (for of HDF) and 5 J/cm^2 UVA (for HEK). DCFDA reacted and immediately observed fluorescence images by confocal microscopy (Nikon, TE-2000, Tokyo, Japan) with excitation / emission at 485 nm / 535 nm. HDF : human dermal fibroblast; HEK : human epithelial keratinocytes; UVA : ultraviolet A.
